# Perceptions of School-Based Telehealth in a Rural State: Moving Forward After COVID-19

**DOI:** 10.5195/ijt.2021.6370

**Published:** 2021-06-22

**Authors:** Susan Skees Hermes, Jade Rauen, Shirley O'Brien

**Affiliations:** 1 Department of Occupational Science and Occupational Therapy, College of Health Sciences, Eastern Kentucky University, Richmond, Kentucky, USA

**Keywords:** COVID-19, Occupational Therapy, School-based Practice, Telehealth, Telepractice, Telerehabilitation

## Abstract

The purpose of this study was to discern the barriers faced by school-based clinicians, chiefly occupational therapists (OTs) and speech-language pathologists (SLPs) who provided telehealth in a primarily rural state during an unexpected declaration of a state of emergency in response to the COVID-19 pandemic. Survey results found the major barriers to implementation of telehealth services to be lack of practitioner training, a lack of access to technology for students, and concerns that the quality of intervention might not be equivalent to in-person service delivery. This article discusses both the benefits and barriers to providing telehealth services in school-based practice and offers considerations for future studies on this topic.

Over the last decade, increased access and availability of technology has led to a growing body of research designed to evaluate the efficacy of implementing interventions in school-based practice via telecommunication. Prior to the COVID-19 pandemic, relatively few school-based occupational therapists and speech-language pathologists employed telehealth. The pandemic required unprecedented changes to the lives of children and families, to school systems, and to school-based practice, precipitating rapid transitioning to a virtual context. As of May 2021, at the writing of this article, restrictions to in-person instruction remained in place in many schools, and practitioners continued to provide virtual interventions for children.

The purpose of this study was to identify barriers to the practice of school-based occupational therapists and speech-language pathologists providing telehealth in a primarily rural state during an unexpected declaration of a state of emergency. The state demographics are primarily rural with most communities under 40,000 in population and only two with higher density communities that range from 325,000 to over 600,000 residents (World Population Review, 2021)

Professionals in occupational therapy (OT) and speech-language pathology (SLP) consider telehealth as an accepted mode of service delivery and it is endorsed by the American Occupational Therapy Association (AOTA, 2018) and the American Speech-Language Hearing Association (ASHA, 2016). Such practice is referred to as telehealth by AOTA and telepractice by ASHA (2016); and often as telerehabilitation or telemedicine by the American Telemedicine Association (Brennan, et al., 2010). Since the authors of the current study come from an occupational therapy background, for the purposes of this article, the term telehealth will be used. AOTA defines telehealth as “the application of evaluative, consultative, preventative, and therapeutic services delivered through information and communication technology” (AOTA, 2018, p.1). The occupational therapy profession supports telehealth as a service deliver model to achieve many outcomes including occupational performance, participation in activities of daily living, health and wellness, role competence, quality of life, and occupational justice (AOTA, 2018).

Current literature suggests promising evidence for the use of telehealth by the health professions, though technology and lack of physical contact are acknowledged as possible barriers. Telehealth can remove barriers to care for clients who live in remote locales, or in areas with a shortage of clinicians (Campbell et al., 2020; Cason, 2012; Johnsson et al., 2019; Jones et al., 2014). Stakeholders generally express positive feedback about telehealth because it has resulted in improved health outcomes (Camden & Silva, 2021; Sanchez et al., 2019; Tenforde, et al. 2020; Wallisch, et al., 2019). Several studies examined the efficacy of telehealth in achieving client outcomes and performing clinician tasks and found no difference in assessment scores between in-person and telehealth consultations for environmental consultations. Tenforde et al., (2020) reported an overall positive perception of telehealth service delivery for the pediatric population. Families were satisfied with the delivery of health care services, especially that telehealth enabled them to observe their children in their natural environments, thus supporting their daily engagement.

School-based research in a variety of specific populations has similarly shown telehealth to be effective. In one case-study, school-based telehealth sessions improved the skills of children with handwriting deficits (Criss, 2013). Another study found online programs delivered through telehealth to be as effective as in-person interventions to improve symptoms of developmental dyslexia (Pecina et al., 2019). Little et al. (2018) found occupation-based coaching by means of telehealth improved educational outcomes for students with autism.

As a result of this new wave of evidence, more schools have begun to employ telehealth as a service delivery model. During the 2007-2008 school year, only 7% of school-based health centers utilized telehealth. This number grew to 19% by the 2016-2017 school year giving over one million students in over 1800 public schools access to a school-based health center (SBHC) using telehealth. However, this number represents only 2% of students and nearly 2% of public schools in the United States (Love et al., 2019).

Prior to the COVID-19 pandemic, many states and school systems had shown interest in creating telehealth programs. This prompted studies on the perceptions on benefits of telehealth, and perceived support and barriers to implementation in the school-based practice setting. Yet, professional development needs were explored in a limited capacity, with reticence by clinicians to embrace a changing delivery model. In a 2019 pilot study, practitioners without telehealth experience expressed several perceived barriers: (1) logistics, (2) lack of physical contact, (3) student factors, (4) privacy concerns, (5) difficulty completing student evaluations, and (6) other perceived barriers. The same study found that 42% of respondents expressed an interest in participating in an educational program about telehealth (Rortvedt & Jacobs, 2019). Conditions for success in virtual delivery have been reported to include technical skills, digital literacy, online privacy, ethics, and knowledge of how to use therapeutic skills via a digital context (Camden & Silva, 2021).

## METHODS

A quantitative cross-sectional design was implemented to capture the perceptions of the school-based practitioners and administrators (Creswell & Creswell, 2018). The Department of Education in the researchers' state initiated a survey to better understand the role telehealth could play as a service delivery option in a K-12 rural educational system. A subset of the larger needs assessment survey data comprised the current study, with analysis of three select questions, along with demographics. Institutional Review Board approval was obtained prior to analysis of the select items. Each participant provided consent through a question item before beginning the survey. Survey responses were de-identified prior to data analysis.

## PROCEDURE

This study utilized a portion of a larger needs assessment that was initiated by a state telehealth workgroup to address implementation of telehealth services in rural school-based practice.

The survey, conducted early in the COVID-19 pandemic, served as a part of the master schedule that had been developed by the greater taskforce prior to the pandemic. The Qualtrics Software platform was used to develop the survey and disseminated it via email to the superintendent of every school district in the state as well as stakeholder organizations for practitioners to be distributed to school-based providers. Superintendents, district representatives, and school-based clinicians from across a mid-central state were invited to participate. Geographic locations of participants included urban, suburban, and rural school settings. The survey was made available virtually from May 2020 to June 2020 to increase participation despite the chaotic state of the educational system. Each participant provided consent through a question item before beginning the survey.

## PARTICIPANTS

This study analyzed the responses of 96 individuals who represented 54 unique counties in the state.

**Figure 1:**
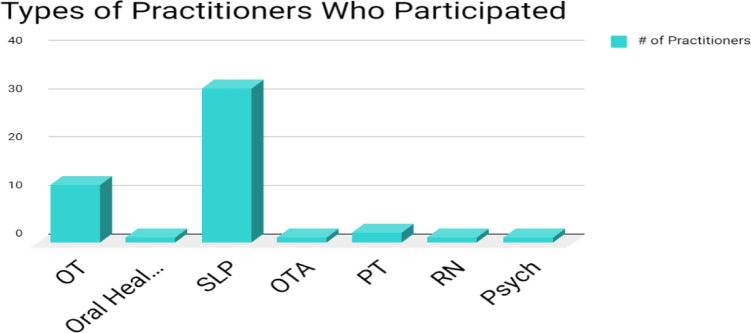
Types of Practitioners Who Participated in the Survey

Fifty respondents were direct service providers and 46 were school district representatives. Of those direct service providers, speech-language pathologists (n=32) and occupational therapists made (n=12) up the most participants with at least one response from several other school-based disciplines (see Figure 1).

## RESULTS

Of the 167 total responses to the original survey, 96 respondents answered the questions related to telehealth usage prior to and during the COVID-19 pandemic (57.5%). All 167 gave their consent to participate in the survey. Some questions had more responses than others as certain questions did not pertain to some participants.

**Figure 2:**
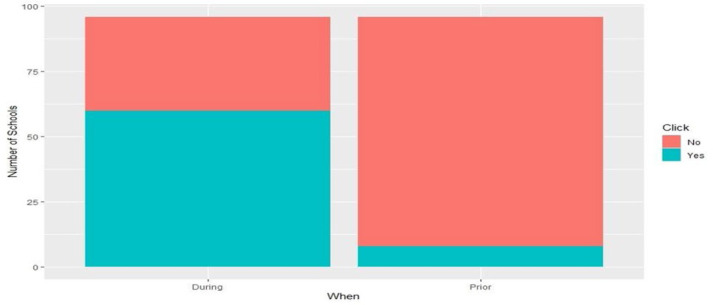
Use of Telehealth in School-Based Practice Prior to and During the COVID-1 Pandemic

The following are some of the participant demographic trends. Of those surveyed, 64.8% of participants served children from a rural community, 20.4% served urban communities, and 14.8% served suburban communities. Participants identified use of telehealth in their school-based practice prior to and during the COVID-19 pandemic. Of the 96 respondents, only 8.3% reported using telehealth in their school district prior to the pandemic. During the pandemic, 60 of the 96 participants (62.5%) utilized telehealth in their practice. A chi-square test of independence was performed to examine the relation between the utilization of telehealth prior to and during the pandemic. The relation between these variables was significant, χ^2^ (1, N = 61.571) = 8.9, p <.001 (see Figure 2).

Participants identified barriers to implementation of telehealth during the COVID-19 pandemic by choosing all that may apply to their situation. The options included data security for student confidentiality related to the Family Education and Privacy Act (FERPA)/Health Information Portability and Accountability Act (HIPAA), billing, technology for providers, technology for students, workflow, space, reimbursement, training, costs (i.e., service and equipment), internet access for students, jurisdiction, access to providers to deliver telehealth, quality concerns that telehealth is not equivalent to in-person care, negative provider attitudes towards telehealth, parental consent, collaborative needs for telehealth (i.e., schools, provider, students, parents), provider personal choice (i.e., childcare, immune compromised), initiation, and other. The top identified barriers were practitioner training (49%), student technology needs (45%), and quality concerns that telehealth was not equivalent to in-person treatment (40%). This data set also was found to be statistically significant, χ^2^ (18, N = 96) = 216, p = .0029. Results are provided in Table 1.

**Table 1:** Identified Barriers to Implementation of Telehealth during the COVID-19 Pandemic

Barrier	% who identified barrier
Training	49.0%
Technology for Students	44.8%
Quality Concerns	39.6%
Unsure how to initiate	30.2%
Security	30.2%
Collaboration Needs	28.1%
Consent	27.0%
No Need	26.0%
Technology for Providers	22.9%
Cost	20.1%
Workflow	19.8%
Attitudes toward Telehealth	11.5%
Reimbursement	13.5%
Access for Providers	11.5%
Space	8.3%
Other	4.1%
Jurisdiction	4.1%
Provider Choice	3.1%

Due to the data collection method, differences in barriers between rural and urban settings were hard to establish, but overall barriers were consistent with previous barrier studies conducted in rural settings (Campbell, et al., 2019; Johnsson, et al., 2019). Future studies should look at this barrier in greater depth, as general technology questions may not address the complex issues at hand in US households. The pandemic has forced practitioners and school systems to face questions of bandwidth, number of devices versus number of students in the home, adequate cameras and positioning, financial means for wireless internet, and geographic availability of wireless internet.

## DISCUSSION

Survey results revealed that prior to the pandemic, many districts were not utilizing telehealth, a trend that is seen nationwide. In addition to the provider data, the larger needs assessment gathered input on school-based health centers (SBHC). These trends also inform allied health providers' practice. While the percentage of SBHCs using telehealth did grow from 7% in the 2007-2008 school year to 19% in 2016-2017 school year, this still only accounts for 1800 public schools. The 1800 public schools that had access to an SBHC that is using telehealth represents only 2% of students and 2% of public schools in the United States (Love et al., 2019).

The main barriers to implementation were found to be practitioner training, student technology needs, and quality concerns that telehealth is not equivalent to in-person intervention. Training and quality concerns as perceived barriers were consistent with previous literature (Camden & Silva, 2021; Rortvedt & Jacobs, 2017). According to Nissen and Brockvelt (2016), education is a critical factor necessary to promote successful implementation of telehealth in clinical practice. In occupational therapy, new practitioners are required to gain a background knowledge of telehealth according to ACOTE Standard B. 4.15 (American Occupational Therapy Association, 2018). Many current practitioners did not have any formal education on telehealth and did not know where to start. Therapy practitioners should learn about various strategies for health care implementation within new opportunities for service delivery that are cost effective and meet client needs. While there is no current literature discussing best practices for teaching occupational therapy practitioners telehealth practices in the school-based setting, a recent study on nurses demonstrated promising results. This study established the effectiveness of a training on telehealth competencies to develop nurses' knowledge of telehealth. In each learning team, telehealth knowledge and self-efficacy significantly increased 6-10 weeks after the training (van Houwelingen et al., 2020). Thus, seasoned practitioners can learn telehealth concepts and use them to implement telehealth.

The third barrier addressed, a fear that telehealth does not provide the same quality of intervention as in-person care, can be addressed through educational programs on telehealth. Studies have shown telehealth to be effective in school-based settings; and education on the benefits of telehealth should minimize this barrier and address the fears of practitioners (Criss, 2013; Sanchez et al, 2019; Wallisch, 2019). This discussion has occurred in concert with the delivery of online learning versus traditional in-person instruction. The needs of professionals are paramount, along with online pedagogy, learning needs, and quality assurance (O'Brien, 2017; Shisley, 2020). School-based interventions have traditionally been delivered in-person. The movement to telehealth has shifted public perception and discussion to the complexity of use of technology along with practitioners yearning for foundational training within the context of student growth and learning.

## IMPLICATIONS

When COVID-19 is a distant memory, the lived experience of virtual schooling will live on. While we see the difficulties with prolonged use, millions of dollars of funding have been put into supporting tele-education and telehealth within 2020 to 2021. In the future, it would make sense to use those purchases to supplement “out of building” learning. This needs assessment shows a need for training and education that addresses practitioners' perception of preparedness, allowing them to switch service delivery with little notice (Dahl-Poplizio, et al., 2020). Additionally, school districts that plan to utilize state Non-Traditional Instruction (NTI) must, in collaboration with parents, develop strategies to serve children with Individualized Educational Programs (IEPs) during NTI to help them maintain progress toward learning goals. Occupational therapists and other school-based providers can advocate for the use of the technology purchased during COVID-19 to utilize telehealth's benefits, bringing specialized practitioners to students who otherwise would not be able to receive those services.

In 2017, the graduation rate across the state in which this survey was conducted for all students was 90.3%. In that same year, only 76% of students with an IEP graduated, even when adjusted for a five-year timeline (Kentucky Department of Education, 2019). As more schools begin to take part in NTI for out-of-building events after the COVID-19 pandemic, students with disabilities need to receive the needed attention so that they do not fall behind. The forced adoption of the virtual context due to the global pandemic and local levels of states' emergencies which alter policy enforcement and legal ramifications still calls for best practice guidance (Smith et al., 2020). Our efforts are timed with other researchers attempting to bridge the need to determine the role of previous supports and barriers and how the current pandemic provides needed information by exploring occupational therapists' perceptions.

During out-of-building sessions, it may be not only an ethical imperative, but legally necessary, to develop a plan for these students on the basis of the Individuals with Disabilities Education Act and the Rehabilitation Act of 1973 (Clark et.al., 2020; IDEA, 2004), and compliant with privacy laws that include FERPA & HIPAA (AOTA, 2018; Cohn & Cason, 2019; Fischbach, 2019). Although the use of technology for service delivery had a relatively slow start to widespread adoption by clients, providers, and payers, many efforts have laid a foundation since 2012 with position statements and definitions of terminology (AOTA, 2018; Cason, 2012; Cason & Jacobs, 2014; Cohn & Cason, 2019; Fischbach, 2019) even though different disciplines and practice settings use the terms “telehealth,” “telepractice,” and “telerehabilitation” interchangeably.

Data driven decision making can assist with accountability shifting modes of service delivery and effectiveness (Schaff, 2015; Smith-Foley, 2020). Best practice for telehealth dictates that the selection should be in the best interest of the client, with the following factors addressed in the choice for service delivery models: (1) telehealth knowledge, skills, and competency of the provider, (2) telehealth regulatory or requirements of the practice setting/state, (3) the client(s)’ context and environment, (4) the client(s)’ condition complexities, and (5) the intervention components for telehealth delivery (AOTA, 2018; Cohn & Cason, 2019; Fischbach, 2019). Use of telehealth requires continuous monitoring to assure it remains the best fit for the client's needs and that progress is being made toward identified goals. Clinicians have had concerns regarding their own comfort with the use of telehealth, reliability of the connections, the quality of the interventions compared to the familiar traditional in-person delivery, and the support of administration, funding sources, and the patients’ satisfaction (Campbell et al., 2019; Dahl-Poplizio et al, 2020). These factors may have previously been slowing the adoption of telehealth. As a global push towards telehealth in response to COVID-19, a plethora of new evidence has begun to surface, and it can be expected that much more will be presented and formalized in the future (Camden & Silva, 2021; Dahl-Popolizio et al., 2020; Smith, et al., 2020; Smith-Foley, 2020). Prior to this pandemic, limited literature had been published regarding widespread use of telehealth across pediatric and school-based populations. States looking forward to telehealth and school-based practice should seek new evidence that addresses the specific populations, diagnoses, and ages can benefit the most from a telehealth service delivery model.

## LIMITATIONS

This study was limited by sample size and total representation of the entire state as only 56 out of 171 total school districts responded to the survey. Additionally, the survey was conducted during the first months of the COVID-19 pandemic; an unprecedented transition to forced remote learning with little to no notice for many schools and practitioners. The set-up of the survey became a limitation to data analysis. Therefore, a future survey may be developed with data specialists to create a manageable set of raw data.

## CONCLUSION

The use of telehealth in school-based settings is an emerging service delivery model across disciplines. This study provides feedback from practitioners regarding the barriers for implementing telehealth during out-of-classroom experiences. The literature revealed that telehealth can remove access barriers to school-based practitioners, specifically in areas where therapists and specialty practitioners are limited and travel times are limiting factors. The variety of barriers and responses from practitioners showcased the complexity of remote learning, especially in populations that are forced to engage in NTI without notice or training. In the wake of the COVID-19 pandemic, a wave of new literature will be available for reference to further support and refine the scope of practice appropriate for school-based telehealth services. Policy creators at the state and school district level could benefit from a scoping review of new literature to clarify “during the pandemic” changes and evidence-based best practice.
